# Application of double low-dose mode in left atrial-pulmonary venous computed tomography angiography

**DOI:** 10.1038/s41598-023-48973-x

**Published:** 2023-12-07

**Authors:** Changjiang Zhang, Wei Zhang, Kaihu Shi, Jingya Chen

**Affiliations:** 1 Affiliated Hospital of Integrated Traditional Chinese and Western Medicine, Nanjing University of Chinese Medicine, Nanjing, China; 2 Affiliated Hospital of Nanjing University of Chinese Medicine, Nanjing, China

**Keywords:** Cardiology, Health care

## Abstract

This study adopted a 256-slice iCT scanner with the double low-dose mode in left atrial-pulmonary venous computed tomography angiography (CTA) and explored its effect on image quality. 120 patients were included and randomly classified into the Observation group and Control group. Patients in the Control group underwent routine left atrial CTA, while patients in the Observation group performed a double low-dose mode. Other scanning parameters were consistent in the two groups. The Full model-based iterative reconstruction (MBIR) technique was applied to fulfill image reconstruction in observation group. Continuous variables, ordered categorical variables were analyzed by statistical test. The CT values of left atrial in the Observation group were significantly higher than those in the Control group. The exposure doses (ED) and iodine intake were lower in the Observation group, as compared to the Control group. The left atrial-pulmonary venous CTA with the 256-slice iCT scanner in a double low-dose mode can reduce the ED of radiation and iodine contrast while providing high quality images. Comparatively, the ED in the Observation group was reduced by 13% compared with the control, and the iodine intake was reduced by approximately 33%.

## Introduction

Cardiac multidetector computed tomography (MDCT) is commonly applied in patients with atrial fibrillation (AF) prior to radiofrequency ablation (RFA)^1^. It can depict the structure, morphology, opening diameter, and other information about the left atrium (LA) and pulmonary veins (PV). Importantly, it can also give a view of thrombus in the LA appendage (LAA), helping guide formulation of operative procedures^2^. MDCT is particularly effective to display the three-dimensional structure and position of the LV and PVs. CT angiography (CTA) has become of growing utility in AF patients prior to RFA^3^. However, the effects of ionizing radiation and contrast agents on kidney function are inevitable, and there has been a growing emphasis on the safety of CTA^4^. Many investigations have been focused on the scan strategies that can both meet the clinical requirements for image quality and minimize the ED of radiation and contrast agent. The radiation dose of CT scan is dependent on multiple factors, including the tube voltage, tube current, scan range, pitch, and layer thickness. Reduction in the tube current or voltage is a major approach to decrease the radiation dose for CT scan. The former is easy to obtain and applied more in routine physical examination of the chest. Nevertheless, excessive current reduction can lead to increased image noise and then decreased image quality. To the contrary, reduction in tube voltage can further enhance the vascular contrast while decreasing the radiation dose, showing the feasibility of double low-dose technique based on low-tube voltage and low-dose iodine contrast. MBIR technique^5^ can reduce the radiation dose by decreasing the tube voltage, the tube current, or the product of time and tube current. Notably, it will not reduce the image spatial resolution, but further decrease image noise and improve image quality. Fewer current reports focus on the use of MDCT in left atrial-pulmonary venous CTA, especially on the image quality under a double low-dose mode^6,7^.

The current study aims at discussing the effect of the double low-dose technique (low-tube voltage and iodine contrast) combined with the MBIR technique on the quality of left atrial-pulmonary venous CTA images, so as to provide theoretical evidence for clinical application.

## Methods

### Patient data

**Figure 1:**
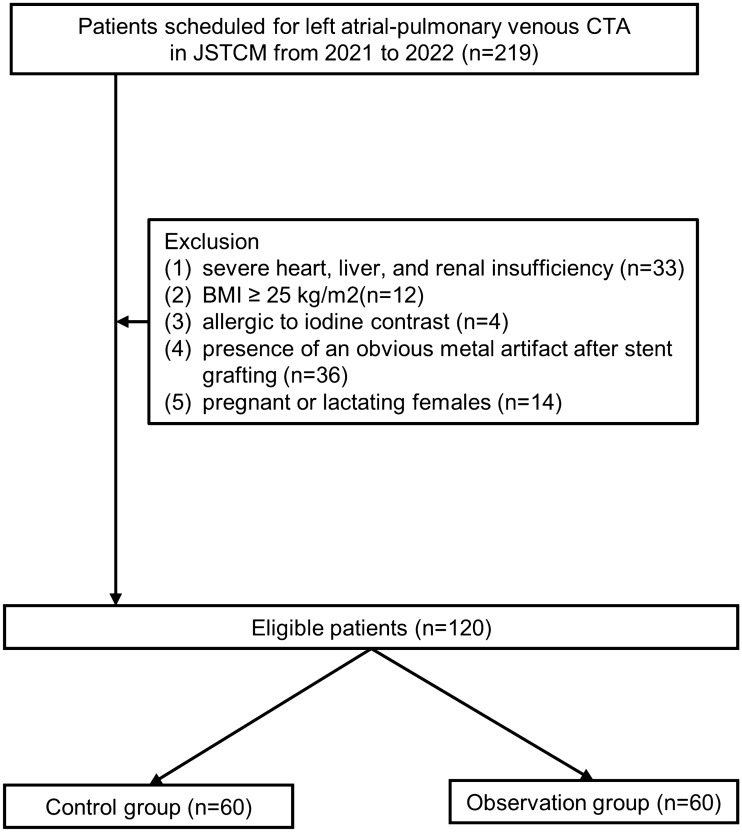
The scheme of study design.

This is a prospective study with the approval of the ethics committee of affiliated hospital of Nanjing university of Chinese medicine (Y2018CX52). All participants were fully informed of the procedures and signed informed consent before participation. We confirm that all methods were carried out in accordance with relevant guidelines and regulations. Patients scheduled for left atrial-pulmonary venous CTA due to suspected or known cardiovascular disease or AF were enrolled in this study, who were first categorized by body weight and then randomly assigned to one of the corresponding subgroups. 219 consecutive patients were included in this study, and their gender, age, heart rate, and body mass index (BMI) were collected. Most patients are admitted to hospital with atrial fibrillation. Exclusion criteria: (1) severe heart, liver, and renal insufficiency (n = 33); (2) BMI ≥ 25 kg/m^2^ (n = 12); (3) allergic to iodine contrast (n = 4); (4) presence of an obvious metal artifact after stent grafting (n = 36); (5) pregnant or lactating females (n = 14). The participants were randomly assigned to the Observation group (n = 60) and Control group (n = 60). The study design scheme was shown in Fig. 1.

### Examination methods

All participants were informed of the procedure by an experienced physician before examination, and proper respiratory training was given to make the patients relaxed. CT scans were acquired on a Philips Brilliance 256-slice iCT scanner and spital scanning was performed. The scan ranges from tip to bottom of lung. Nonionic contrast agent iohexol (350 mg/mL) was injected through the right median cubital vein using a double-syringe power injector at a rate of 5 mL/s. The Observation group received a dose of 0.7 ml/kg, while the Control group received a dose of 1 mL/kg. Consistently, 30 ml normal saline was injected in both groups with the same rate after iohexol injection. Scanning parameters were set as below: tube voltage: 100 kV for the Observation group and 120 kV for the Control group; detector width: 128 × 0.625; pitch: 0.27; layer thickness: 0.9 mm; layer spacing: 0.45 mm; rotation time: 330 ms. The tube current was auto-regulated. Contrast track-and-trigger technique was applied to depict the regions of interest (ROI) in the LA regions, with the trigger threshold set as 100 HU. The images were processed for image reconstruction with the MBIR technique and then transferred onto a Philips post-processing workstation.

### Image post-processing and subjective/objective assessment

The reconstructed images were transferred onto a Philips workstation for post-processing. All images were assessed by two senior radiologists with respect to the LA (8 and 12 years of cardio-thoracic radiology experience, respectively), LAA, and PVs using the volume rendering (VR) and multiple planar reformation (MPR) approaches. Any discrepancy was resolved by discussion.

The subjective criteria for assessing image quality adopted a 5-point scoring system^4^: 1-point: low image quality, unclear anatomical structure, severe artifacts, not acceptable for diagnosis; 2-point: low image quality, unclear anatomical structure, and moderate artifacts; 3-point: moderate image quality, moderate artifacts, clear anatomical structure in the majority, acceptable for diagnosis; 4-point: good image quality, clear anatomical structure, minor artifacts; 5-point: high image quality, no artifact, well-defined anatomical structure. Images scored > 3 points were acceptable for clinical diagnosis.

The objective assessment of image quality was performed as below. The LA, the opening of the PV trunk, the LAA cavity, and the pectoralis major were selected as ROIs from the images of cross-sections, and corresponding enhanced CT values were obtained. ROI areas were confined to 3–8 cm^2^, and the ROIs were draw away from calcification, vessel wall, and the columnar crest in LAA. Image noise was calculated as the standard deviation (SD) of the enhanced CT value of ascending aortic root. Image noise was assessed by signal–noise ratio (SNR) and contrast-to-noise ratio (CNR) with the following formula: SNR = average vascular CT value/SD, and CNR = (vascular CT value − muscle CT value)/vascular SD.

Radiation doses, volume CT dose index (CTD), and dose length product (DLP) were automatically generated by the CT scanner and recorded. Effective radiation dose (ED) was calculated as: ED = DLP × k, where k represents the conversion factor, which is 0.017 mSv/(mGy cm) in chest CTA. Additionally, iodine intake from the contrast was measured as: I (g) = contrast concentration (350 mg/ml) × contrast dose (ml)/1000.

### Statistical analysis

SPSS 22 was applied for statistical analysis. All data were initially processed for normality test. The continuous variables meeting normal distribution were analyzed by an independent-sample t test; otherwise, Mann–Whitney U test was used. Dichotomous categories and their proportions were tested by a Chi-square test or a Fisher exact probability test. Intraclass correlation coefficient (ICC) was measured to assess the consistency between the subjective and objective results. P < 0.05 indicated a difference with statistical significance.

### Ethical approval

All procedures performed in studies involving human participants were in accordance with the ethical standards of the institutional and/or national research committee and with the 1964 Helsinki declaration and its later amendments or comparable ethical standards. This article does not contain any studies with animals performed by any of the authors.

## Results

### Subjective assessment

The subjective scores in both the Observation and Control groups were non-normally distributed, and the mean score (95% CI) was 4 (3.72–4.26) and 4.24 (3.96–4.51), respectively. There was no significant difference between the two groups (Mann–Whitney U test: Z = − 1.303, p = 0.193). See Table 1 for more details.

**Table 1:** Baseline characteristics of patients.

Group	Age (years)	Male/female	Weight (kg)	HR	BMI
Observation (n = 60)	72.8 ± 8.1	33/27	60.1 ± 8.8	65.7 ± 10.9	22.47 ± 1.77
Control (n = 60)	69.6 ± 11.2	36/23	58.1 ± 8.5	66.2 ± 9.08	21.82 ± 2.03
P	0.2	0.39	0.39	0.39	0.237

### Objective assessment

**Figure 2:**
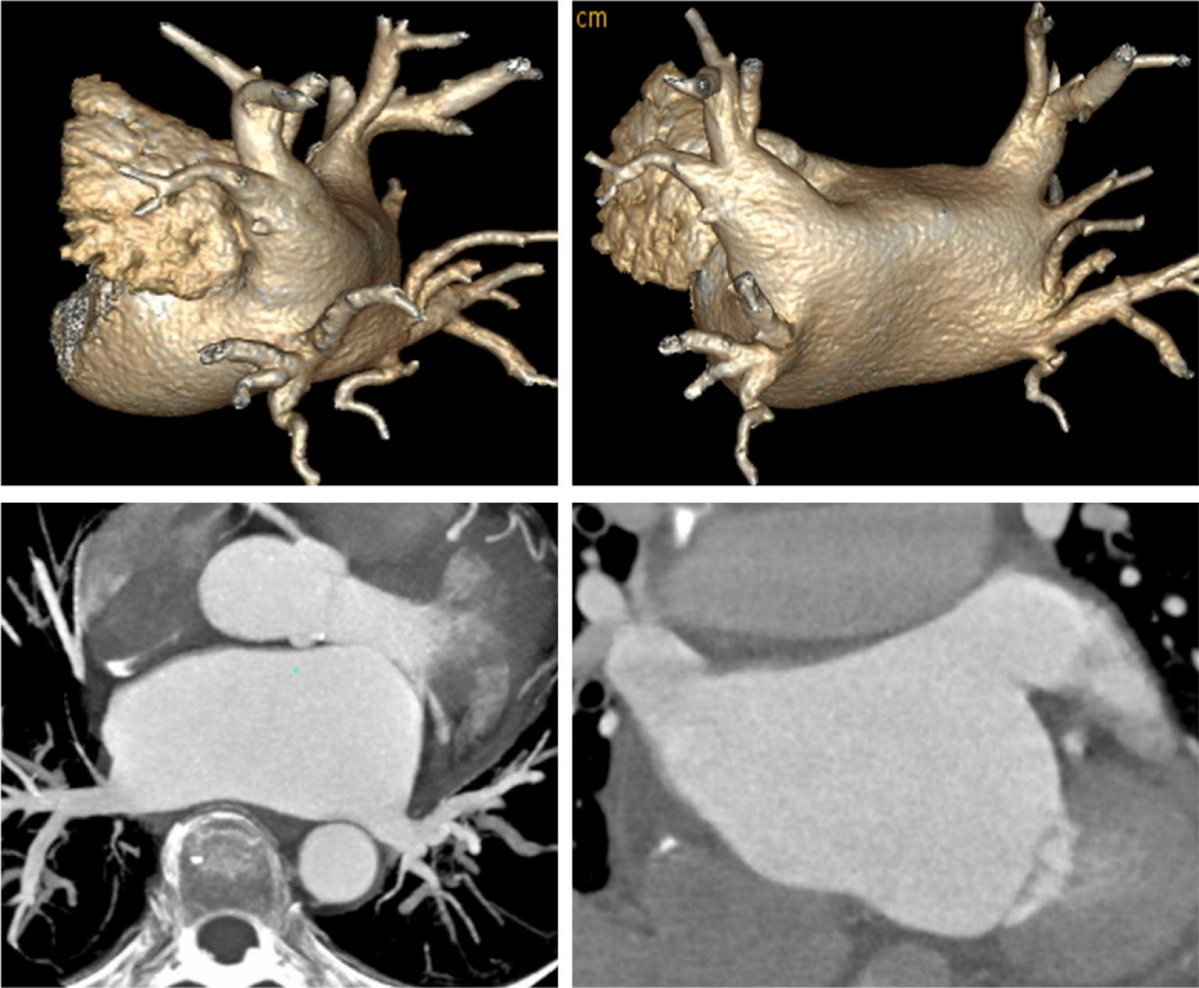
Images of 65 years old male in observation group. BMI = 23.6, tube voltage was 100 kV, iodine contrast agent dose was 17.5 g. VR and MPR images showed that the left atrium, pulmonary vein and left atrial appendage were well depicted, and the image quality score was 4 points.

**Figure 3:**
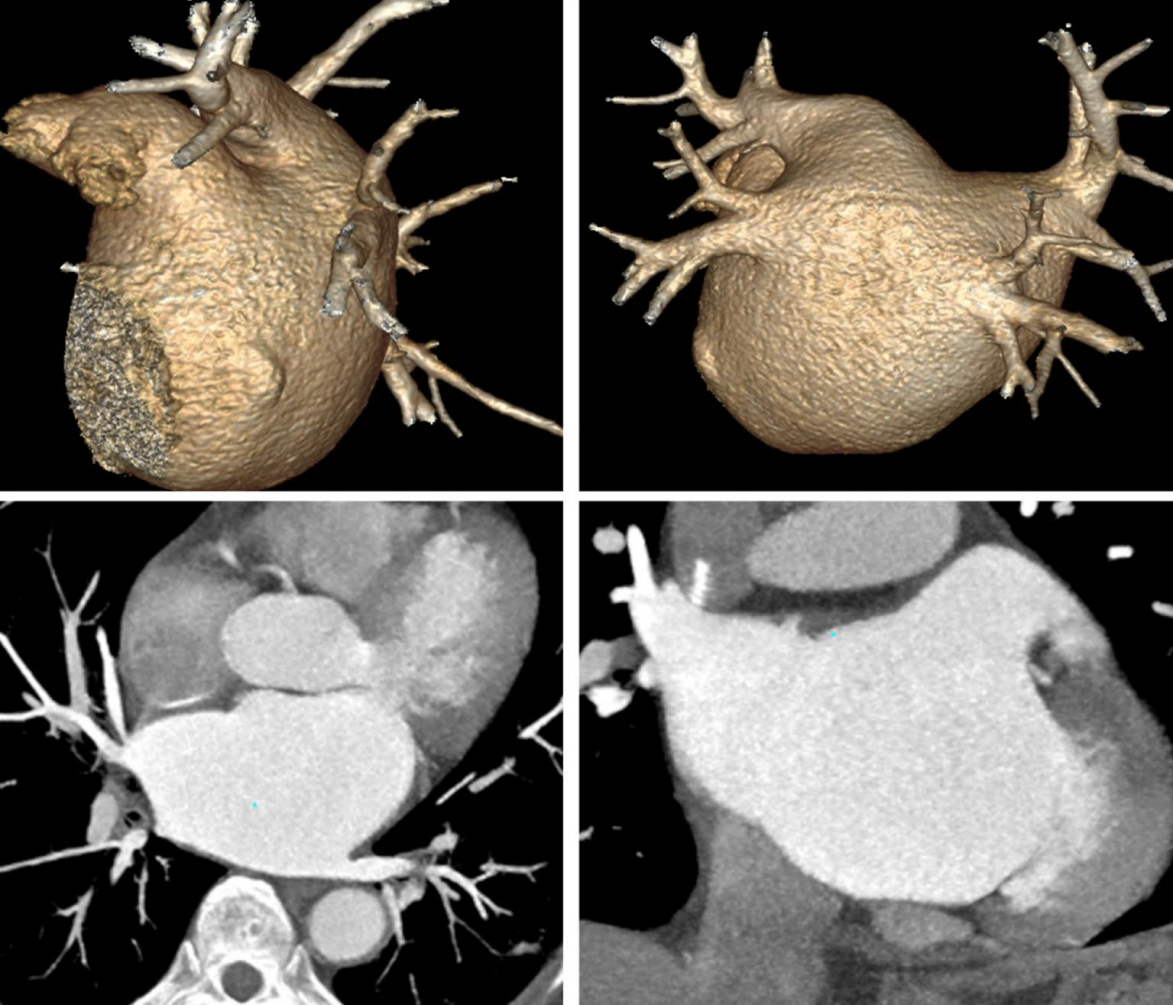
Images of 52 years old female in control group. BMI = 24.9, tube voltage was 120 kV, iodine contrast agent dose was 20 g, VR and MPR images showed that left atrium, pulmonary vein, left atrial appendage, etc. were clearly developed, and the image quality score was 5 points.

The enhanced CT values of ROIs, including the opening of the PV trunk, mid LA, and LAA cavity, were significantly higher in the Observation group than the Control group (p < 0.05, Table 2). Besides, the SNR and CNR in the Observation group were slightly lower than those in the Control group, and the differences were not statistically significant (Table 3). The image quality of both groups can meet the requirements of clinical diagnosis (Figs. 2, 3).

**Table 2:** The measured CT value in the observation and control groups.

Group	Pulmonary vein (HU)	Left atrium (HU)	LA appendage (HU)	Aortic root (HU)	Pectoralis major (HU)
Observation (n = 60)	401.5 ± 41.1	443.6 ± 53.1	424.8 ± 69.1	389.7 ± 41.8	140.9 ± 26.1
Control (n = 60)	374.9 ± 35.4	418.1 ± 36.8	387.8 ± 39.7	358.3 ± 47.7	125.7 ± 26.2
P	0.01	0.035	0.014	0.009	0.028

**Table 3:** Objective scoring in the observation and control groups.

Group	SNR	CNR	CTD	DLP	ED (mSv)	I (g)
Observation (n = 60)	6.8 ± 0.9	4.2 ± 1.1	21.8 ± 5.7	320.8 ± 87.7	5.5 ± 1.1	14.3 ± 2.1
Control (n = 60)	6.9 ± 0.9	4.5 ± 0.7	24.2 ± 8.8	368.1 ± 74.5	6.3 ± 1.2	21.1 ± 3.1
P	0.43	0.13	0.24	0.04	0.04	< 0.01

### Radiation dose and iodine intake

The ED was (5.5 ± 1.1) mSv in the Observation group and (6.3 ± 1.2) mSv in the Control group, while the iodine intake was (14.3 ± 2.1) g in the Observation group and (21.1 ± 3.1) g in the Control group. Both the differences were of statistical significance (p < 0.05). Comparatively, the ED in the Observation group was reduced by 13% compared with the control, and the iodine intake was reduced by approximately 33%.

## Discussion

This study shows that the image quality obtained by low voltage, low contrast agent and MBIR technique can meet the diagnostic requirements. The method can effectively reduce the radiation dose and renal function changes caused by contrast medium. This double low-dose mode technique is useful in medical practice.

AF is the most common atrial arrhythmia in clinic^8^. It is usually accompanied by reduction in left ventricular systolic function, leading to blood stasis in the LAA and a high susceptibility to cardiogenic emboli formation. In the meantime, dislodging of the emboli can lead to occlusion of the systemic circulation. According to statistics, approximately 60% of the cardiogenic strokes are derived from AF^9,10^. Pulmonary vein ablation is generally applied for the treatment of FA cases that are hard to be managed by drugs, and LAA occlusion is a viable option to prevent the risk of developing stroke induced by dislodging of emboli^1^. An accurate knowledge of the anatomic and pathological conditions of the LA, PVs, and LAA before operation is of paramount clinical significance to guide procedures, shorten the operation time, monitor postoperative complications, and perform a dynamic follow-up.

Depending on the high temporal and spatial resolutions of MDCT, it has been increasingly applied in clinic, especially in the cardiovascular screening with multiple indications^3,5,11^. MDCT can depict the opening width of the PVs and LAA, and depict the thrombi in the LA system. In addition, it can also provide the three-dimensional locations of the PVs and LAA relative to the esophagus. Therefore, MDCT has promising application prospect in preoperative examination of AF patients^7^. With the extensive application of the CTA technique, the ionizing radiation and iodine contrast have attracted increasing attention for their potential risk of harm to the patients^12,13^. Glands and organs that are sensitive (partially sensitive) to X-irradiation, such as the thyroid and gonads, have an increasing risk of developing cancer when exposed to high-dose ionizing radiation on CT scans. Except the ionizing radiation, the iodine contrast used by CTA also greatly affects the renal function, probably resulting in renal dysfunction^14^. In this context, there is an urgent need to look for new scan strategies that can be effective with a low dose of radiation and iodine contrast while maintaining the image quality^15^. To this end, double low-dose scan technique arises^16,17^.

In the present study, left atrial-pulmonary venous CTA was fulfilled in 120 patients. Both the Observation and Control groups obtained satisfactory CTA images, which clearly showed the relative positions of the LA, PVs, LAA, and the LA system to the esophagus, and the image quality was acceptable for clinical diagnosis. The CT values of the ROIs were significantly higher in the Observation group than the Control group based on the objective assessment, but no statistical differences were noted by subjective assessment. The result demonstrated that the double low-dose scan strategy does not affect the image quality (within the acceptable range for clinical diagnosis) while reducing the radiation and contrast doses.

Reduction in tube voltage can enhance the photoelectric effect of X-ray and increase the X-ray attenuation associated with the iodine-containing tissues, resulting in higher CT value of iodine contrast and enhanced contrast of the atria and PVs with the surrounding tissues^12,14^. Consistently, our study found significant higher CT values in the Observation groups than the Control group. Nevertheless, the reduction in tube voltage simultaneously decreases the X-ray's penetrating power, leading to increased image noise and decreased image quality^18,19^. The MBIR technique provides an optimized statistical model composed of multiple factors, including X-ray beam's width, detector pixel size, and voxel size. It can provide accurate image information, and reduce the noise caused by reduction in tube voltage. With this technique, the decrease in CT value following low-dose contrast administration can be improved to some extent, which makes the double low-dose technique theoretically feasible^20^. Moreover, this study found lower SNR and CNR in the Observation group, which might be due to the increase in image noise caused by low voltage^21–23^. However, there were no statistical differences between the two groups. This indicates that MDCT with the MBIR technique can decrease the image noise from double low-dose scans and thus increase the image quality.

There are limitations to the study. First, the sample size is small, and high-BMI patients common in clinic are not included in this study. The scan strategy for some individuals requires further exploration. Second, the dose of contrast used in this study adopts a fixed dosing schema and is not individualized by BMI. Thirdly, AI has been widely used to improve the image quality and does reduction nowadays^16,24^, it will be performed and compared in the future studies. Lastly, the lack of individual-specific approaches for different patients were not taken in this study, which may have affected the results, these approaches will be performed in clinical settings in the following research.

To conclude, left atrial-pulmonary venous CTA with the double low-dose technique can reduce the exposure dose of radiation and iodine contrast while maintaining the image quality within an acceptable range for clinical diagnosis, showing a safety profile.

## Data Availability

The datasets used during the current study available from the corresponding author on reasonable request.
